# Social listening – revealing Parkinson’s disease over day and night

**DOI:** 10.1186/s12883-020-02024-4

**Published:** 2021-01-04

**Authors:** Hui Zhang, Fanwen Meng, Xingyu Li, Yali Ning, Meng Cai

**Affiliations:** 1 UCB Pharma, Room 02, Floor 28, Raffles Office Building, 268 Tibet Middle Road, Huangpu District, Shanghai, 200003 China; 2 Department of Neurology, Zhongshan Hospital Fudan University, Shanghai, China

**Keywords:** Parkinson’s disease, Nocturnal symptoms, 24-h symptom management, Social listening, Dopamine agonist

## Abstract

**Background:**

Nocturnal symptoms in Parkinson’s disease are often treated after management of daytime manifestations. In order to better understand the unmet needs of nocturnal symptoms management, we analyzed the characteristics and burden of nocturnal symptoms from patients’ perspectives and explored their changes over time. Overall symptoms (occurring at day or night) were collected to compare whether the unmet needs related to nocturnal symptoms and to overall symptoms are different.

**Methods:**

We used a Social Listening big-data technique to analyze large amounts of Parkinson’s disease symptoms in dialogues available from social media platforms in 2016 to 2018. These symptoms were classified as either overall symptoms or nocturnal symptoms. We used share of voice (SOV) of symptoms as a proportion of total dialogues per year to reflect the characteristics of symptoms. Negative sentiment score of symptoms was analyzed to find out their related burden.

**Results:**

We found the SOV for overall motor symptoms was 79% and had not increased between 2016 and 2018 (79%, *p* = 0.5). The SOV for non-motor symptoms was 69% and had grown by 7% in 2018 (*p* <  0.01). The SOV for motor complications was 9% and had increased by 6% in 2018 (*p* <  0.01). The SOV of motor symptoms was larger than non-motor symptoms and motor complications (*p* <  0.01). The SOV of non-motor symptoms was larger than motor complications (*p* <  0.01). For nocturnal symptoms, 45% of the analyzed PD population reported nocturnal symptoms in 2018, growing by 6% (*p* <  0.01). The SOV for nocturnal-occurring motor symptoms was higher than most non-motor symptoms. However, non-motor symptoms had the higher increases and evoked higher negative sentiment regardless of whether they occurred during the day or night. For symptoms that can occur at either day or night, each nocturnal symptom was rated with a higher negative sentiment score than the same symptom during the day.

**Conclusions:**

The growing SOV and the greater negative sentiment of nocturnal symptoms suggest management of nocturnal symptoms is an unmet need of patients. A greater emphasis on detecting and treating nocturnal symptoms with 24-h care is encouraged.

**Supplementary Information:**

The online version contains supplementary material available at 10.1186/s12883-020-02024-4.

## Background

Parkinson’s disease (PD) is the most common movement disorder of the central nervous system [1]. It is estimated that PD affects 1–2% of the population above 65 years of age worldwide [2]. In China, the overall prevalence has been estimated at 190 per 100,000 individuals, with an overall incidence of 362 per 100,000 person years [3]. PD is characterized by both motor and non-motor system manifestations. Motor symptoms of resting tremors, rigidity and bradykinesia are the cardinal signs of the disease. Along with the disease progress and nonphysiological stimulation of striatal dopamine receptors especially long-term levodopa therapy, motor symptoms could be unstable presented as motor complications including motor fluctuations, dyskinesias [4]. While the 24-h nature of symptoms is known, focus on the management of nocturnal disturbances is comparatively low [5–7], but studies have reported as many as 96–98% of PD patients being affected by disabling nocturnal symptoms [5, 7]. Nocturnal symptoms may be classified into three classes using the Revised Parkinson’s Disease Sleep Scale (PDSS-2): 1) “motor problems at night”, such as tremor, early morning dystonia; 2) “PD symptoms at night”, such as pain, cramps, hallucinations and immobility; and 3) “disturbed sleep”, such as insomnia, nocturia, difficulty staying asleep, and general poor sleep quality [8]. In practice, all classes of nocturnal symptoms may affect quality of life (QoL) and result in disruptions to normal sleep. Some daytime symptoms may also be exacerbated or produced by nocturnal symptoms [5]. Patients may experience symptom fluctuations associated with the wearing-off of their levodopa dose overnight or in the early morning [9, 10]. Nocturnal symptoms tend to be exacerbated with disease progression and are associated with a worse prognosis [11, 12]. However, these symptoms occur outside of healthcare professionals’ contact hours with patients. In addition, patients may not be able to describe their nocturnal symptoms accurately or may not consider the symptom part of their PD pathology. Further, symptoms such as rapid eye movement sleep behavior disorder (RBD) require caregiver or observer reports. This relative inaccessibility to clinicians may be part of the reason of a diminished focus on nocturnal symptoms [12, 13]. To better understand the unmet needs of nocturnal symptom management from patients’ perspective, a patient-centric approach that allows for a freer expression of certain concerns may be useful.

In recent years, the examination of social media data to better understand a population has become possible through a technique called Social Listening (SL), in which publicly available information can be investigated to determine what topics are being discussed and what attitudes the contributing population holds towards the subjects of discussion [14]. Aside from creating a large dataset, which strengthens the conclusions that can be drawn from the data, SL has the advantage of collecting data from outlets which patients use voluntarily, and it imposes no additional burden to patients as surveys or other studies might. The technique is also a cost-effective way of collecting a large amount of diverse patient-centric data, which makes SL particularly useful in resource-challenged environments. The extent to which different symptoms are discussed in such public forums can be reasonably assumed to be a proxy for the issues of concern – and therefore unmet needs – of the patients discussing them.

Thus, to understand the unmet needs of nocturnal symptoms from the patients’ perspective, we used SL to analyze large amounts of patient-doctor and patient-patient interaction data in 2016 to 2018 available from clinical practitioners’ consulting platforms, online forums and PD bulletin-board systems in China. We sought to know the characteristics and the burden of nocturnal symptoms from patients’ perspectives. In addition, although dopaminergic therapy, especially levodopa, has improved the treatment of PD symptoms significantly over the past decades, symptoms that persist through day and night and affect non-dopaminergic systems are still of large and of growing concern. In this study, we also explored whether there were any changes in participants’ needs over time by comparing data across 3 years. As a contrast, overall symptom data, including symptoms occurring during the day, were also collected in the study. Even though the primary focus in usual clinical practice is often on motor symptoms, particularly those affecting daytime activities, we investigated whether the unmet needs of nocturnal symptoms and overall symptoms are different from patients’ perspective, which is useful in devising a strategy for symptom management.

Because SL is a relatively new technique, no national guidelines concerning the ethics of SL in a research setting exist in China. Upon registering on the websites that were used as data sources for this study, users gave their e-consent to agree with the collection and use of their provided data. The use of that data for this study was considered legally and ethically proper as it was obtained from publicly accessible platforms, did not contain individually identifiable information, and was analyzed only at the group level.

## Methods

### Dataset

We employed a custom web crawler written in the Python programming language [15] to crawl and collect dialogues from PD-related bulletin-board system (BBS) forums and e-consulting platforms between January 2016 and December 2018. The consulting platforms and bulletin-board systems are listed in Supplementary Table 1. Bulletin boards were included to provide data from an extra-clinical setting and offer a large sample of patients in less-structured environments. Dialogues collected through these platforms covered approximately 40,000 patients and 3000 healthcare practitioners. Raw data collection included 60,000 dialogues, approximately 10,000 of which came from bulletin boards. Only dialogues that featured at least one PD symptom were included for analysis. When narrowed by this criterion, a total of 15,119 dialogues (2016: 7524, 2017: 5198, and 2018: 2397) were included for analysis. In all cases, dialogues were in Mandarin, and where possible demographic information such as the age of the patients was also collected.

The raw dialogues lacked standardization of speech and contained grammatical errors. To address this, we used a Bigram semantic model that corrected for typing errors and double negation. After correction, named entity recognition using a Bidirectional Encoder Representations from Transformers (BERT) [16] Long Short-Term Memory (LSTM) [17] Conditional Random Field (CRF) [18] model was used to analyze the sentences for content according to context and the presence of keywords in the categories “motor symptoms”, “non-motor symptoms”, “motor complications” and “nocturnal symptoms”. The keywords initially used in the model were selected by the authors to reflect common terms used in the literature and clinical settings. Representative keywords for the motor symptoms included ‘tremor’, ‘rigidity’, ‘bradykinesia’; non-motor symptoms were RBD, ‘sensory disorder’; motor complications were ‘dyskinesia’, ‘motor fluctuation’, etc. Motor symptoms such as difficulty walking, reduced facial expression and stiffness refer to symptoms which were a result of the PD itself, whereas complications for example, peak-dose dyskinesia, reflect the known effects of dopaminergic medications for treating PD. Nocturnal symptom keywords were those giving any indication that the symptom occurred at night. The full set of keywords is available in Supplementary Table 2. Not all keywords used in the model returned results from the dataset; those that did not were not further considered in the analysis. We used share of voice (SOV), which represents the proportion of total dialogues per year accounted for by each symptom category, to reflect the characteristics of symptoms from patients’ perspectives. A negative sentiment score for the symptoms was analyzed to investigate their related burdens on patients.

### Statistical analyses

Independent samples t-tests were conducted to compare SOV of each symptom group based on the data reported from 2016 to 2018. Further t-tests were done to compare the SOV of overall motor to non-motor symptoms, and non-motor symptoms to motor complications. Changes in the SOV of individual symptoms within each symptom group were also compared using independent samples t-tests.

Negative sentiment analysis was also conducted on the dataset. For negative sentiment and PD symptoms, only dialogues with sentiment words and PD symptoms were included. Negative sentiment words were graded by severity (see Table 1). Negative sentiment words were in Mandarin; for the list of words deemed acceptable for a given sentiment see Supplementary Table 3. For a given symptom, the average negative sentiment was calculated in the following manner:

$$ \left(\left(\left(\%\mathsf{of}\ \mathsf{suspicious}\ \mathsf{dialogues}\right)\times \kern0.37em \mathsf{1}\right)+\left(\left(\%\mathsf{of}\ \mathsf{anxiety}\ \mathsf{dialogues}\right)\times \kern0.37em \mathsf{2}\right)\dots +\left(\left(\%\mathsf{of}\ \mathsf{sorrowful}\ \mathsf{dialogues}\right)\times \kern0.37em \mathsf{6}\right)\right)/\mathsf{100} $$

**Table 1:** Sentiment word weighting

Sentiment word	Negative weight
Suspicion	1
Anxiety	2
Fear	3
Agony	4
Anger	5
Sorrow	6

Using this method, which has been widely adopted in social listening research, a single score combining both the weighting and proportion of reported negative sentiment was created for comparison.

## Results

### Patients

Demographic information was available for only 2895 of the 15,119 dialogues analyzed. In that data, most patients were over the age of 50 (86%), with patients between 60 and 70 years old forming the largest group (33.1%). The mean age (±standard deviation) of patients was 63 years (±13.4). Demographic data is represented in Table 2.

**Table 2:** Demographics

Age Group (years)	Participants (n, %)
< 50	405 (14.0%)
50–60	586 (20.2%)
60–70	957 (33.1%)
> 70	947 (32.7%)
Total:	2895 (100%)

### Share of voice and change of overall symptoms

**Fig. 1:**
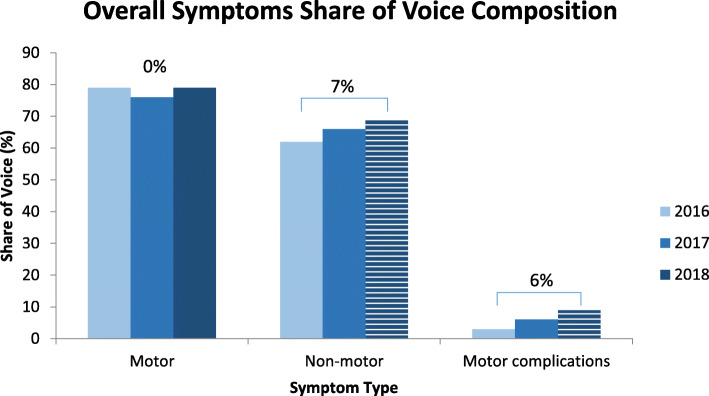
Overall Symptoms Share of Voice Composition Across a Three-Year Period. Percentages are the size of compounded annual growth rate (CAGR) comparing 2016 to 2018; striped bars indicate statistically significant changes in CAGR at the level of *p* <  0.01

Symptom SOV representing the proportion of dialogues mentioning a given symptom, is depicted in Fig. 1. In line with their role as cardinal symptoms, motor symptoms had the highest SOV. Non-motor symptoms also had a significant SOV. The SOV for occurrence of motor symptoms was 79% in 2018, with no growth from 2016 (*p* = 0.5). The SOV for non-motor symptoms was high at 62% in 2016, reaching 69% in 2018, a statistically significant 7% increase from 2016 (*p* <  0.01). The SOV for occurrence of motor complications was 9% in 2018, with 6% growth from 2016 (*p* <  0.01). Motor symptoms maintained a significantly larger SOV than non-motor symptoms and motor complications in every year, (*p* <  0.01), as did the SOV of non-motor symptoms compared to motor complication SOV (*p* <  0.01). See Table 3, Table 4 and Table 5 for comparison.

**Table 3:** Motor Symptoms vs. Non-motor Symptoms Across a Three-Year Period

Year	Motor symptom	Non-motor symptom	***p***-value
2018 (*N* = 2397)	79%	69%	< 0.01
2017 (*N* = 5198)	76%	66%	< 0.01
2016 (*N* = 7524)	79%	62%	< 0.01

**Table 4:** Non-motor Symptoms vs. Motor Complications Across a Three-Year Period

Year	Non-motor symptom	Motor complications	*p*-value
2018 (*N* = 2397)	69%	9%	< 0.01
2017 (*N* = 5198)	66%	6%	< 0.01
2016 (*N* = 7524)	62%	3%	< 0.01

**Table 5:** Motor Symptoms vs. Motor Complications Across a Three-Year Period

Year	Motor symptom	Motor complications	*p*-value
2018 (*N* = 2397)	79%	9%	< 0.01
2017 (*N* = 5198)	76%	6%	< 0.01
2016 (*N* = 7524)	79%	3%	< 0.01

For the details of major motor and non-motor symptoms, see Table 6 and Table 7, respectively. Canonical motor symptoms, like tremors (52.7%), stiffness (29.5%), difficulty walking/slow movement (24.5%), and problems turning over (13.9%) were the most commonly mentioned motor symptoms. Gait postural instabilities (79.3%), falling (16.6%), problems turning over (15%), difficulty walking/slow movement (8.9%) and stiffness (8.0%) all showed increases across the sample period (*p* <  0.01) with reduced facial expression decreasing by 18.2% (*p* <  0.01). Changes in tremor, unclear enunciation and speech disorder were all non-significant.

**Table 6:** Overall Motor Symptoms Composition Across a Three-Year Period

Type	Symptom	2016	2017	2018	2016–2018 CAGR^a^	***p***-value
Tremor	Tremor	55.6%	51.9%	52.7%	−2.6%	0.03
Rigidity	Stiffness	25.3%	24.6%	29.5%	+ 8.0%	< 0.01
	Problems turning over	10.5%	11.6%	13.9%	+ 15.0%	< 0.01
Bradykinesia	Difficulty walking/slow movement	20.7%	19.2%	24.5%	+ 8.9%	< 0.01
	Reduced facial expression	4.5%	3.0%	3.0%	−18.2%	< 0.01
	Unclear enunciation	3.0%	2.2%	2.8%	−2.0%	0.35
	Speech disorder	0.8%	0.7%	1.0%	+ 14.4%	0.25
Postural Instability	Falling	7.3%	7.8%	9.9%	+ 16.6%	< 0.01
	Gait postural instabilities	1.6%	2.7%	5.1%	+ 79.3%	< 0.01

^a^*CAGR* Compounded annual growth rate

**Table 7:** Overall Non-motor Symptoms Composition Across a Three-Year Period

	Symptom	2016	2017	2018	2016–2018 CAGR^**a**^	*p*-value
Sleep and Sensory Disorders	Pain	24.4%	25.2%	25.8%	+ 2.9%	0.15
	Frequent urination	7.5%	8.6%	11.8%	+ 25.8%	< 0.01
	Insomnia	6.2%	6.3%	8.9%	+ 20.0%	< 0.01
	RBD	3.9%	5.1%	7.4%	+ 37.3%	< 0.01
	Poor sleep quality	5.4%	5.6%	5.7%	+ 2.4%	0.34
	Excessive Daytime Sleepiness	3.8%	4.1%	5.6%	+ 20.9%	< 0.01
	Numbness	4.9%	4.2%	5.0%	+ 1.4%	0.44
	Spasm	3.7%	3.8%	3.6%	−1.6%	0.43
	Olfactory disorder	0.9%	1.0%	2.5%	+ 64.5%	< 0.01
Cognitive and Psychiatric Disorders	Depression	6.1%	7.0%	11.4%	+ 36.2%	< 0.01
	Hallucinations	7.2%	9.1%	10.0%	+ 18.1%	< 0.01
	Dementia	5.7%	6.3%	9.1%	+ 26.6%	< 0.01
	Anxiety	2.4%	3.3%	6.9%	+ 70.6%	< 0.01
	Slow reaction	2.0%	2.0%	3.4%	+ 31.4%	< 0.01
	Other psychiatric issues	1.2%	1.1%	1.3%	+ 4.7%	0.39
Autonomic Disorders	Constipation	7.9%	8.4%	12.1%	+ 23.9%	< 0.01
	Other gastrointestinal dysfunctions	6.8%	8.3%	8.1%	+ 9.5%	0.06
	Drooling	4.9%	4.8%	5.9%	+ 9.6%	0.08

^a^*CAGR* Compounded annual growth rate

For non-motor symptoms, pain (25.8%), constipation (12.1%), frequent urination (11.8%), and depression (11.4%) had the largest SOV. The largest increases in symptom mentions were in anxiety (70.6%, *p* < 0.01) and olfactory disorders (64.5%, *p* < 0.01), followed by RBD (37.3%, *p* < 0.01). In general, there was a larger increase in the mention of non-motor symptoms over the reported period compared to the change of SOV for motor symptoms.

### Share of voice and change of nocturnal symptoms

Nocturnal symptoms, which included all motor and non-motor symptoms and motor complications that occurred at night, can be seen in Table 8. Overall, they had a SOV of 45% in 2018, growing by a compounded annual growth rate (CAGR) of 6% from 2016 (*p* < 0.01). In 2018, the SOV for individual nocturnal symptoms ranged from 0.8 to 16%. Rigidity, specifically difficulty turning over (16%), insomnia (8.9%) and night tremors (8.7%), had the highest SOV of all nocturnal symptoms in 2018. Difficulty breathing (52.2%) and morning pain (51.0%) had the highest increases in SOV over the 3-year period but remained low in overall SOV. Of interest was the substantial SOV held by sleep-related disturbances (insomnia, RBD, poor sleep quality and frequent nocturia), which corresponded to the fact that symptoms with high SOV tend to result in disruptions of normal sleep. Overall, nocturnal symptoms had a strong tendency to increase over the reported period. Only poor sleep quality and nocturnal spasm did not significantly increase over time (*p* = 0.34 and *p* = 0.38, respectively).

**Table 8:** Nocturnal Motor and Non-motor Symptoms Composition Across a Three-Year Period

Symptom	2016	2017	2018	2016–2018 CAGR^**a**^	*p*-value
Overall Nocturnal Symptoms	39%	41%	45%	+ 6.0%	< 0.01
Rigidity/difficulty turning over	11.9%	12.8%	16.0%	+ 16.0%	< 0.01
Insomnia	6.2%	6.3%	8.9%	+ 20.0%	< 0.01
Night tremor	7.0%	6.8%	8.7%	+ 11.2%	0.02
RBD	3.9%	5.1%	7.4%	+ 37.3%	< 0.01
Nocturnal pain	5.3%	5.9%	7.3%	+ 17.3%	< 0.01
Poor sleep quality	5.4%	5.6%	5.7%	+ 2.4%	0.34
Excessive Daytime Sleepiness	3.8%	4.1%	5.6%	+ 20.9%	< 0.01
Frequent nocturia	2.9%	3.6%	5.3%	+ 35.6%	< 0.01
Hallucinations	1.6%	2.1%	2.5%	+ 26.9%	0.03
Morning pain	0.6%	1.0%	1.4%	+ 51.0%	< 0.01
Nocturnal spasm	1.1%	1.0%	1.2%	+ 8.2%	0.38
Difficulty breathing	0.3%	0.5%	0.8%	+ 52.2%	0.02

^a^*CAGR* Compounded annual growth rate

### Negative sentiment of overall symptoms and nocturnal symptoms

**Fig. 2:**
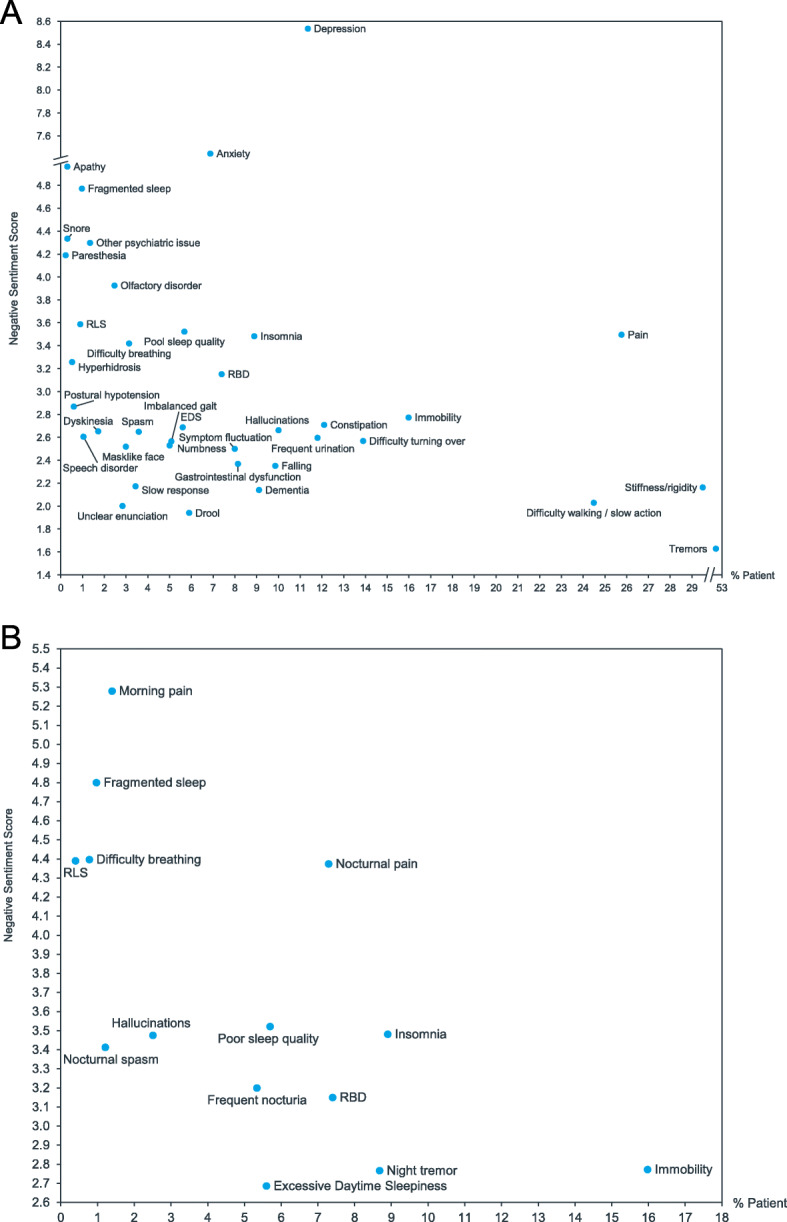
% SOV for PD symptom vs. Negative Sentiment Score for PD Symptom. Panel A: All symptoms; Panel B: Nocturnal symptoms

**Fig. 3:**
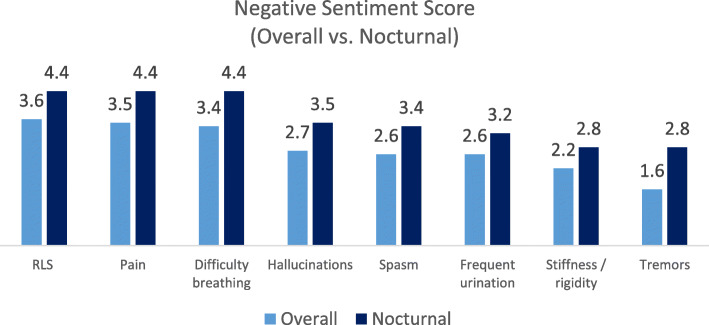
Comparison of sentiment for PD symptoms when occurring at any time and occurring nocturnally. Only symptoms that do not exclusively occur nocturnally are able to be compared

Figure 2 shows a graph of symptoms and their corresponding negative sentiment. In Panel A, all PD symptoms that featured as keywords are shown with their associated negative sentiment score. The symptom group with the highest SOV (motor symptoms) had the lowest average negative sentiment. Non-motor symptoms had higher scores of negative sentiment on average, and a greater range of ratings. Psychiatric symptoms like depression (8.5) and anxiety (7.4) scored highest. See Table 9 for sentiment scores for selected overall motor and non-motor symptoms with higher SOV or sentiment scores for their category. Panel B, and Table 10, show the nocturnal symptom scores, some of which have no corresponding daytime equivalent (e.g. insomnia). Morning pain (5.3), fragmented sleep (4.8), and nocturnal pain (4.4) were particularly high scoring. For symptoms that can occur at either night or day, each nocturnal symptom was rated with a higher negative sentiment score than the same symptom across night and day combined, regardless of the type of symptom (motor, non-motor, or motor complication). Figure 3 shows the comparison of negative sentiment for the symptoms that can occur at either day or night. For the complete list of symptoms and their respective sentiment scores, see Supplementary Table 4.

**Table 9:** Overall sentiment scores for selected motor and non-motor (^a^) symptoms

PD Symptom	Score
Depression^a^	8.5
Anxiety^a^	7.4
Apathy^a^	5.0
Olfactory disorder^a^	3.9
Restless Legs Syndrome^a^	3.6
Pain^a^	3.5
Difficulty breathing^a^	3.4
Immobility	2.8
Hallucinations^a^	2.7
Excessive Daytime Sleepiness^a^	2.7
Difficulty turning over	2.6
Frequent urination^a^	2.6
Spasm ^a^	2.6
Falling	2.3
Stiffness/rigidity	2.2
Tremors	1.6

**Table 10:** Sentiment scores for nocturnal motor and non-motor (^a^) symptoms

Nocturnal Symptom	Score
Morning pain^a^	5.3
Fragmented sleep^a^	4.8
Restless Legs Syndrome^a^	4.4
Nocturnal pain^a^	4.4
Difficulty breathing^a^	4.4
Insomnia^a^	3.5
Poor sleep quality^a^	3.5
Hallucinations^a^	3.5
Nocturnal spasm ^a^	3.4
Frequent nocturia^a^	3.2
RBD^a^	3.1
Night tremor	2.8
Nocturnal Immobility/stiffness/rigidity	2.8
Excessive Daytime Sleepiness^a^	2.7

## Discussion

This study represents an attempt to understand the unmet needs of nocturnal symptoms from patients’ perspective. We explored the frequency and the burden of negative sentiment of nocturnal symptoms in a large dataset (15,119 dialogues) using a social listening technique. SOV of motor symptoms was the highest in nocturnal symptoms. For overall symptoms, SOV of motor symptoms was larger significantly than non-motor symptoms and motor complications. The results indicated motor symptoms were still patients’ most concerning problems. The most prominent nocturnal motor symptom was rigidity, a condition whereby patients have restricted or no movement, often to the extent that they cannot turn over or get out of bed. This is consistent with the profile of PD, as rigidity is one of the most difficult symptoms to adequately alleviate [19].

The SOV of nocturnal symptoms increased over the 3 years studied. Compared with the highest SOV, of nocturnal motor symptoms, the SOV of nocturnal non-motor symptoms increased across the 3-year period. Amongst nocturnal non-motor symptoms, insomnia, pain, and RBD featured with high SOV and CAGRs in our data. Such nocturnal non-motor symptoms may arise for multiple reasons. First, in contrast to motor symptoms which are improved by dopaminergic drugs, nocturnal symptoms may become more prevalent with disease progression [7, 20] but are less- or un-improved by dopaminergic drugs. Thus, attention on the improvement of non-motor symptoms is growing [21]. Second, interplay exists between motor and non-motor symptoms [10]. For example, insomnia and nocturnal pain are associated with nocturnal motor symptoms [22]. Lower than optimum dopaminergic drug dosages may cause insufficient control of nocturnal akinesia, tremors, which then exacerbate nocturnal non-motor symptoms [5, 23, 24]. In addition, undesired effects of dopaminergic drugs, or the use of inappropriate medication or dosages may also play a role. The dosage and pharmacokinetics of levodopa are associated with the development of complications [21]. Reliance on higher doses of levodopa rather than using adjunctive medications or modifying delivery regimens may exacerbate or induce nocturnal symptoms [5]. However, it is important to note that no data to explain why SOV changed over time were collected in this study. As such, although we speculate that growth in SOV represents an unmet need and increased patient focus on nocturnal symptoms, this question should be addressed in detail in a future work. There is limited research on nocturnal symptoms to date, and our investigation should be considered both tentative and exploratory for these reasons.

Although SOV of motor symptoms was highest in our data, non-motor symptoms evoked higher negative sentiment scores no matter whether they occurred in the daytime or at night (see Fig. 2). Whilst any symptom that impairs daily functioning can be expected to evoke negative sentiment, non-motor symptoms like depression may be particularly debilitating. One reason is that many non-motor symptoms are not reported or enquired about. They are often not recognized by the consulting clinician and may thus go untreated [6, 13]. Further, the pathological and biochemical mechanisms for many non-motor symptoms involve dopaminergic and non-dopaminergic systems (i.e., noradrenergic, serotoninergic and cholinergic systems). Despite being the major therapy for Parkinson’s disease, the effects of dopaminergic drugs on improving non-motor symptoms remains at least partially unclear [25, 26]; non-motor symptoms may be more treatment-resistant as well as more troubling. It is worth noting that for symptoms occurring over day and night, negative sentiment toward PD symptoms was more pronounced when they occurred nocturnally (see Fig. 3). Control over nocturnal symptoms may not be as good as daytime symptoms due to nocturnal wearing-off, or reduced attention on nocturnal symptom control. By disrupting sleep initiation or maintenance, these symptoms may exacerbate daytime symptoms as well as being uncomfortable during the night, and thus elicit a higher negative sentiment. Further work should assess this hypothesis.

### Implications for managing nocturnal symptoms

With an increasing SOV and a higher negative sentiment of nocturnal symptoms from the patients’ perspective, it is important for healthcare providers to spend more time focusing on the management of these symptoms. Considering the nocturnal inaccessibility of patients, approaches to measure nocturnal symptoms could be more varied. Some wearable devices could provide a continuous objective measurement (COM) to track symptoms over 24 h [27–29], which is good for clinicians seeking to evaluate 24-h symptoms objectively. Communication through various technologies may be beneficial for healthcare providers and patients alike. Increased use of patient-doctor consulting platforms, or even specialized mobile applications for chronic disease management [30] could be utilized to enhance nocturnal symptoms detection and treatment for patients with PD.

Besides these options,24-h continuous treatment is important. Firstly, the replacement of lost dopamine with dopaminergic drugs in 24 h is necessary for the management of motor symptoms alone – regardless of whether these occur nocturnally or during the daytime. Adjunctive medications [31–33], infusion systems such as DuoDopa (carbidopa/levodopa) [34], continuous dopamine delivery treatments like the transdermal rotigotine patch [35–39], and oral extended release versions of dopamine agents [40, 41] have shown significant promise in ameliorating wearing-off effects and symptom fluctuations, in nocturnal as well as daytime symptoms. In a randomized, placebo-controlled study (the RECOVER trial, quality score, 93%), patients with unsatisfactory control of early morning motor symptoms were evaluated on the PDSS-2 and UPDRS III. The study demonstrated that rotigotine could significantly improve early morning motor symptoms and nocturnal symptoms [35], comparable to levodopa continuous infusion [42]. The rotigotine patch may thus be recommended for the management of nocturnal symptoms [43]. In addition, deep brain stimulation (DBS) is another option which can be kept active over a 24-h period. However, to date the proven benefits of DBS are limited to motor symptoms and DBS is most commonly restricted to use in patients with relatively advanced disease [43, 44]. Secondly, for symptoms which can’t be improved by dopaminergic drugs, especially for non-motor symptoms, there is no standard pharmacological treatment at present. Non-motor symptoms should be improved based on careful assessment of triggering or contributing factors and consideration of other factors, including economic influences, local availability of the drug, local drug approval, the treating physician’s experience and judgment and so on [26]. Non-pharmacological treatments are helpful in conjunction with 24-h drug treatment. Improved sleep hygiene and cognitive-behavioral therapy may be useful for insomnia [22]. Regular exercise and physical therapy can also assist with issues such as joint rigidity and flexed posture [33]. Focused education on symptoms for both patients and families or caregivers is highly recommended. The adoption of non-pharmacological therapies alongside pharmacological treatment early in the disease course is also recommended [45]. The importance of effective multi-specialty management for patients with PD should not be underestimated.

### Limitations

Although powerful, utilizing a social listening technique has some limitations. First, the relative lack of full demographic information constrains the conclusions that can be drawn from the data – general conclusions about the reporting PD population may be valid, but not conclusions about specific groups of PD patients. Further, whilst the candid nature of the interactions makes more information accessible, the available details are constrained by what patients recall, lending itself to potential inaccuracies and skewed data. An ability to further identify and segment patient populations will be an important target in future studies. This study should be considered an early attempt to begin to address these issues.

## Conclusions

Our social listening analysis showed that nocturnal symptoms of PD had a significant and growing SOV and are accompanied by higher negative sentiment. A greater emphasis on detecting nocturnal symptoms by approaches such as social media platforms and wearable devices is strongly encouraged. 24-h continuous pharmacological therapy and non-pharmacological treatments are needed.

## Supplementary Information

**Additional file 1.**

**Additional file 2.**

**Additional file 3.**

**Additional file 4.**

## Data Availability

The datasets used and/or analyzed during the current study are available from the corresponding author on reasonable request.

## Abbreviations

PD: Parkinson’s disease
SL: Social Listening
PDSS-2: Revised Parkinson’s Disease Sleep Scale
RLS: Restless legs syndrome
QoL: Quality of life
BBS: Bulletin-board system
BERT: Bidirectional Encoder Representations from Transformers
LSTM: Long Short-Term Memory
CRF: Conditional Random Field
RBD: Rapid eye movement sleep behavior disorder
SOV: Share of voice
CAGR: Compounded annual growth rate
