# Supplementary material for: Social listening – revealing Parkinson’s disease over day and night
Source: BMC Neurol. 2021 Jan 4;21:2. doi: 10.1186/s12883-020-02024-4 (PMC7780378; doi:10.1186/s12883-020-02024-4)
Supplement: Supplementary file 1 — Additional file 1. [file 12883_2020_2024_MOESM1_ESM.docx]

**Supplementary Table 1 – Platforms**

| **Platform category** | **Platform** | **Platform website** |
| --- | --- | --- |
| Consulting platforms | 好大夫 | [www.haodf.com](http://www.haodf.com) |
| Consulting platforms | 春雨医生 | [www.chunyuyisheng.com/](http://www.chunyuyisheng.com/) |
| Consulting platforms | 微医 | <https://www.guahao.com/> |
| Bulletin-board | 帕金森病网 | [www.pohs.net](http://www.pohs.net) |
| Bulletin-board | 帕金森病友会 | parkinson.wohenok.com |
| Bulletin-board | 中华帕金森病友之家 | [http://www.homefpd.com](http://www.homefpd.com/) |
| Bulletin-board | 青年帕金森之家 | [www.yophome.com](http://www.yophome.com) |
